# The Atlanta Medical College

**Published:** 1859-03

**Authors:** 


### EDITORIAL AND MISCELLANEOUS.

### THE ATLANTA MEDICAL COLLEGE.

As will be perceived, by reference to the advertisement, the fifth course of lectures in the Atlanta Medical College will commence on the first Monday in May next.

As our readers are aware, at the termination of the last course of lectures in this Institution, the faculty determined to add four months to the term of instruction, and commenced a *Preparatory* Course of Lectures, demonstrations and examinations on the first of November, to be continued until the last of February. That course has recently terminated, and we have no hesitation in saying that the result of the experiment has been eminently satisfactory to both Faculty and students. Though no pecuniary profit has attached to the additional instruction, the Faculty have cheerfully borne the labor in view of the advantages to those seeking a place in the profession.

We desire to direct attention to the fact that while other Institutions have been clamoring loudly for *senseless* changes (in the name of "Reform") in the American system of Medical education, hoping to enlist the American Medical Association in their crusade against the Atlanta Medical College, (a formidable competitor,) that Institution has been furnishing the most conclusive evidence of her sincere desire to elevate the standard of medical education, by increasing her course of instruction from four to eight months annually, without requiring any additional fee from the student.

We mean again to call attention to the fact that the *intrigue* upon the part of several medical schools, inaugurated previous to the meeting of the American Medical Association in Nashville, though disappointed in the attainment of its object at that time, was developed into Dr. J. R. Wood's

Special Committee report, upon Medical education, at the last meeting of the association in Washington City.

The *present* movement upon this subject, we denounce as originating with the Nashville University, with the view of discrediting the Atlanta Medical College.

The question very naturally arises, whether the Medical Colleges of the United States in the convention proposed by that report, or the American Medical Association in the action recommended for its adoption, will so far degrade and stultify themselves, as to become instruments in the hands of the Nashville school, for the accomplishment of its selfish designs

As to what the American Medical Association may choose to do, in the way of establishing new conditions for those who may hereafter apply for membership or representation we have nothing to say, but we contend that they have no right to institute any Ex Post Facto proceeding, and any such movement will deserve the contempt of the profession and can be of no avail if resisted by those now entitled to representation.

As to what other Medical Colleges, and especially those out of the State, may choose to do in the way of establishing new conditions, upon which alone there shall be an interchange of students, they can pursue their own course, it is to be hoped however for the credit of common sense, that they will adopt some less absurd conclusion than that involed in the primary idea of the latest edition of "Medical Reformers" that an Institution to be recognized as orthodox and legitimate must teach medicine *only* at a particular season of the year, whether it be located on the Pacific Coast, at the mouth of the Mississippi or in new England. The Corporators of the Atlanta Medical College under the authority of the State of Georgia and with the sanction of a sufficient portion of the profession, not only of our own State, but of the *South* generally, to have secured it remarkble success, have established a Summer School in the Southern country, and so long as sustained by the profession within the borders of Georgia Sovereignty, will pursue the even tenor of their way, without regard to attempts at *dictation* from rivals, whether they come directly or indirectly—from Colleges, Conventions or Associations, controlled by unscrupulous men for the advancement of selfish purposes.

We again denounce the *present* movement upon this subject, as under the control of, and instigated by those who are looking more to their own interests than to the elevation of the profession.

Notwithstanding the position announced in the present article, the Faculty of the Atlanta Medical College stand prepared to increase the facilities for Medical Education and to aid in the elevation of the profession by co-operating in any movement holding out a *reasonable* prospect for the accomplishment of these ends, but we assert that there is found in the regular Medical Profession of the United States an amount of science and skill, far exceeding their appreciation upon the part of the people, and in addition, that the Medical men of the United States will compare favorably with those of any country upon the globe.

In conclusion we would add, that the prospect for a large class at the next course, is exceedingly flattering.

